# Association of Single Nucleotide Polymorphisms in Wnt Signaling Pathway Genes with Breast Cancer in Saudi Patients

**DOI:** 10.1371/journal.pone.0059555

**Published:** 2013-03-14

**Authors:** Mohammad Saud Alanazi, Narasimha Reddy Parine, Jilani Purusottapatnam Shaik, Huda A. Alabdulkarim, Sana Abdulla Ajaj, Zahid Khan

**Affiliations:** 1 Genome Research Chair, Department of Biochemistry, College of Science, King Saud University, Riyadh, Kingdom of Saudi Arabia; 2 The Comprehensive Cancer Center at King Fahad Medical City, Riyadh, Saudi Arabia; 3 Family Medicine Department, College of Medicine, King Saud University, Riyadh, Saudi Arabia; Sanjay Gandhi Medical Institute, India

## Abstract

Breast cancer is a complex heterogeneous disease involving genetic and epigenetic alterations in genes encoding proteins that are components of various signaling pathways. Candidate gene approach have identified association of genetic variants in the Wnt signaling pathway genes and increased susceptibility to several diseases including breast cancer. Due to the rarity of somatic mutations in key genes of Wnt pathway, we investigated the association of genetic variants in these genes with predisposition to breast cancers. We performed a case-control study to identify risk variants by examining 15 SNPs located in 8 genes associated with Wnt signaling. Genotypic analysis of individual locus showed statistically significant association of five SNPs located in β-catenin, AXIN2, DKK3, SFRP3 and TCF7L2 with breast cancers. Increased risk was observed only with the SNP in β-catenin while the other four SNPs conferred protection against breast cancers. Majority of these associations persisted after stratification of the cases based on estrogen receptor status and age of on-set of breast cancer. The rs7775 SNP in exon 6 of SFRP3 gene that codes for either arginine or glycine exhibited very strong association with breast cancer, even after Bonferroni's correction. Apart from these five variants, rs3923086 in AXIN2 and rs3763511 in DKK4 that did not show any association in the overall population were significantly associated with early on-set and estrogen receptor negative breast cancers, respectively. This is the first study to utilize pathway based approach to identify association of risk variants in the Wnt signaling pathway genes with breast cancers. Confirmation of our findings in larger populations of different ethnicities would provide evidence for the role of Wnt pathway as well as screening markers for early detection of breast carcinomas.

## Introduction

Breast cancer is a major health concern worldwide and is a leading cause of cancer related death in women [1]. In the Kingdom of Saudi Arabia, it ranks number one in terms of incidence as well as cancer related mortality in females [1]. Although the age-standardized incidence rate for breast cancers in Saudi Arabia is 3.4 fold lower compared to United States, the median age of onset is 47 years, significantly lower than 62 years observed in patients from United States [1]–[3]. Recent studies have indicated breast cancer to be a heterogeneous disease that includes several molecular subtypes based on gene expression pattern [4], [5]. Moreover, several genetic as well as epigenetic alterations in genes encoding proteins that are component of various signaling pathways including Wnt pathway have been implicated in the etiology of breast cancers [6]–[8]. Apart from genetic events leading to the initiation and progression of the disease earlier studies have shown an association of single nucleotide polymorphisms (SNPs) in different genes with an increased risk of breast cancer in different populations [9]–[11]. Despite advances in the treatment resulting in a trend towards better overall survival of the patient, the complete molecular basis of transformation is still unknown.

In the canonical pathway, members of Wnt family of secreted glycoproteins interact with two co-receptors, the Frizzled seven transmembrane receptor, and the low density lipoprotein receptor related protein LRP5/6. Wnt–receptor interactions lead to inhibition of β-catenin phosphorylation by the serine threonine kinase, glycogen synthase kinase-β (GSK-3β) within a large cytoplasmic complex including Dishevelled (Dsh), adenomatous polyposis coli (APC) and Axin [12]. Inhibition of β-catenin phosphorylation impairs its degradation by the ubiquitin/proteosome pathway. This results in accumulation of the uncomplexed cytosolic molecule which translocates to the nucleus and interacts with TCF/LEF to activate target genes such as cyclin D1 and c-myc, known oncogenes which contribute to malignant progression [12]. Constitutively activated Wnt signaling has been shown to be causally involved in large number of human cancers including colorectal, melanoma, gastric carcinoma, hepatocellular carcinoma, prostate, ovarian and breast cancer. APC mutations occur in Familial Adenomatous Polyposis (FAP) and in 85% of sporadic colorectal cancers (CRC). The remaining fraction of CRCs showed a very high incidence of β-catenin alterations consistent with the notion that mutation in APC and β-catenin are mutually exclusive and together account for almost all CRCs [12], [13]. Elevated levels of nuclear and/or cytoplasmic β-catenin have been reported in a large portion of breast tumor tissue samples (60%), although genetic alterations in Axin, APC and β-catenin in breast cancer are extremely rare [14]. Moreover, WNT1, WNT4, AXIN2 and LEF1 are upregulated and high β-catenin activity is significantly correlated with poor prognosis in breast cancer patients [15], [16]. Aberrations leading to autocrine Wnt signaling pathway activation have also been reported in breast cancer cells [17]. Thus, sufficient evidence suggests the involvement of Wnt pathway in the development and progression of human breast cancer. Due to the reported low frequency of mutations in the β-catenin destruction complex genes, we performed this study to examine the association of SNPs in the Wnt pathway genes with predisposition to breast cancer development in Saudi population.

## Materials and Methods

### Study population

A total of 192 blood samples were obtained from King Khalid University Hospital. These encompassed 99 patients with breast cancer and 93 controls with no history of cancer. Breast cancer cases included patients of all age (median age  = 48 years) and stage of the disease. The patient and control population were from Saudi Arabian ethnicity. All controls were age-matched and recruited from physical examinations after diagnostic exclusion of cancer and cancer- related diseases. Blood samples of the cases were obtained before the start of any treatment.

### Ethics Statement

This study was approved by the institutional review board of King Khalid University Hospital. Written informed consent was obtained from all participants.

### DNA extraction

Approximately 3 ml of blood samples were collected in vacutainers containing ethylenediaminetetraacetic acid (EDTA) from all subjects enrolled in the study. Genomic DNA was isolated from blood samples using QIAmp DNA blood mini kit (Qiagen, Valencia, CA, USA) following the manufacturer's instructions. After extraction and purification, the DNA was quantitated spectrophotometrically on NanoDrop 8000 (Thermo Scientific, USA), and its purity examined using standard A260/A280 and A260/A230 ratios.

### SNP selection and genotyping

SNPs in important genes in the Wnt signaling pathway were randomly selected from SNP500cancer project and from previous literature. The protein products of these genes function at different levels of Wnt signaling pathway and genetic as well as epigenetic alterations in them has been reported in various malignancies including breast cancer [13], [14]. A total of fifteen SNPs in Wnt pathway genes were genotyped using TaqMan allelic discrimination assay [18]. For each sample, 20 ng DNA per reaction was used with 5.6 µL of 2X Universal Master Mix and 200 nM primers (Applied Biosystems, Foster City, CA, USA). All genotypes were determined by endpoint reading on an ABI 7500 real time PCR instrument (Applied Biosystems, Foster City, CA, USA). Primers and probe mix were purchased directly through the assays-on-demand service of Applied Biosystems. Five percent of the samples were randomly selected and subjected to repeat analysis as a quality control measure for verification of genotyping procedures. The results were reproducible without any discrepancies.

### Statistical analysis

Genotype and allelic frequencies were computed and checked for deviation from Hardy-Weinberg equilibrium (http://ihg2.helmholtz-muenchen.de/cgi-bin/hw/hwa1.pl). Case-control genetic comparisons were performed using the chi-square test and odds ratios (OR), and 95% confidence intervals (CI) were calculated by Fisher's exact test (two-tailed). Statistical analysis was done using SPSS 16.0 for Windows. We considered p-value of <0.05 as significant. Additionally, Bonferroni's correction was applied for multiple comparison of the 15 SNPs with an α = 0.0033 considered as significant.

## Results

### Association of genetic variants with breast cancer risk

The study group comprised of 99 women with pathologically confirmed breast cancer and 93 age-matched cancer-free controls. Due to the relevance of Wnt signaling pathway in the pathogenesis of several different malignancies including breast cancers, we selected 15 SNPs in eight genes involved in this pathway to assess the association of genetic variation in these genes with risk of breast cancer in Saudi females (Table 1). Homozygous ancestral allele was used as a reference to determine the odds of acquiring breast cancers in relation to the other two genotypes. The genotype distribution of the analyzed SNPs along with the corresponding odds ratio and significance are shown in Table 2. We observed statistically significant association of SNPs in β-catenin (rs4135385), AXIN2 (rs3923087), DKK3 (rs6485350), SFRP3 (rs7775) and TCF7L2 (rs12255372) genes with breast cancer risk. Of these five SNPs, only SFRP3 SNP rs7775 was in the exonic region that codes for either arginine (Arg) or glycine (Gly) amino acid and was highly significant even after Bonferroni's correction.

**Table 1:** Description of SNPs present in Wnt pathway genes that were analyzed.

Gene	SNP ID	SNP location*	Aminoacid/Nucleotide change	Ancestral allele*
APC	rs459552	NC_000005.9, 112176756	Val1822Asp	T
APC	rs454886	NC_000005.9, 112146117	C>T	T
β-catenin	rs13072632	NC_000003.11, 41262444	C>T	C
β-catenin	rs4135385	NC_000005.9, 112176756	A>G	A
AXIN2	rs4791171	NC_000017.10, 63541497	A>G	A
AXIN2	rs11079571	NC_000017.10, 63548681	A>G	G
AXIN2	rs3923087	NC_000017.10, 63549261	A>G	A
AXIN2	rs3923086	NC_000017.10, 63549488	G>T	T
DKK3	rs6485350	NC_000011.9, 12013617	A>G	A
DKK4	rs3763511	NC_000008.10, 42235858	C>T	C
SFRP3	rs288326	NC_000002.11, 183703336	Arg200Trp	G
SFRP3	rs7775	NC_000002.11, 183699584	Arg324Gly	C
TCF4	rs12255372	NC_000010.10, 114808902	G>T	G
LRP6	rs2075241	NC_000012.11, 12291479	C>G	G
LRP6	rs2284396	NC_000012.11, 12274935	C>T	T

* Source – dbSNP, National Center for Biotechnology Information.

**Table 2:** Genotype frequencies of Wnt pathway gene polymorphism in breast cancers and Controls.

Gene	SNP ID	Genotype	BC (n = 99)	Controls (n = 93)	OR (95% CI)	χ^2^ -Value	P*- Value
APC	rs459552	Val	67 (0.677)	60 (0.645)	Ref		
		Val/Asp	30 (0.303)	31 (0.333)	0.867 (0.470–1.597)	0.21	0.64603
		Asp	2 (0.02)	2 (0.022)	0.896 (0.122–6.556)	0.01	0.91344
APC	rs454886	TT	14 (0.141)	13 (0.14)	Ref		
		CT	44 (0.445)	33 (0.355)	1.238 (0.514–2.984)	0.23	0.63385
		CC	41 (0.414)	47 (0.505)	0.810 (0.342–1.921)	0.23	0.63214
β-Catenin	rs13072632	CC	9 (0.091)	10 (0.107)	Ref		
		CT	46 (0.465)	42 (0.452)	1.217 (0.451–3.285)	0.15	0.69810
		TT	44 (0.444)	41 (0.441)	1.192 (0.440–3.228)	0.12	0.72893
β-Catenin	rs4135385	AA	63 (0.637)	72 (0.774)	Ref		
		AG	31 (0.313)	18 (0.193)	1.968 (1.005–3.854)	3.96	**0.04649**
		GG	5 (0.05)	3 (0.033)	1.905 (0.438–8.290)	0.76	0.38359
AXIN2	rs4791171	AA	34 (0.343)	22 (0.344)	Ref		
		AG	44 (0.445)	44 (0.473)	0.941 (0.497–1.782)	0.03	0.85236
		GG	21 (0.212)	17 (0.183)	1.163 (0.522–2.591)	0.14	0.71233
AXIN2	rs11079571	GG	55 (0.555)	45 (0.484)	Ref		
		GA	37 (0.374)	37 (0.398)	0.818 (0.448–1.494)	0.43	0.51361
		AA	7 (0.071)	11 (0.118)	0.521 (0.187–1.453)	1.59	0.20763
AXIN2	rs3923087	AA	45 (0.46)	24 (0.258)	Ref		
		AG	35 (0.36)	50 (0.538)	0.373 (0.193–0.720)	8.82	**0.00298**
		GG	18 (0.18)	19 (0.204)	0.505 (0.224–1.139)	2.74	0.09772
AXIN2	rs3923086	TT	27 (0.273)	16 (0.172)	Ref		
		TG	41 (0.414)	42 (0.451)	0.578 (0.272–1.229)	2.05	0.15268
		GG	31 (0.313)	35 (0.377)	0.525 (0.239–1.151)	2.62	0.10568
DKK3	rs6485350	AA	39 (0.394)	28 (0.301)	Ref		
		AG	47 (0.475)	43 (0.462)	0.785 (0.415–1.485)	0.56	0.45600
		GG	13 (0.131)	22 (0.237)	0.424 (0.183–0.983)	4.08	**0.04333**
DKK4	rs3763511	CC	70 (0.707)	70 (0.753)	Ref		
		CT	26 (0.263)	23 (0.247)	1.130 (0.589–2.169)	0.14	0.71220
		TT	3 (0.03)	0	7.000 (0.355–138.02)	2.94	0.08650
SFRP3	rs288326	AA	8 (0.081)	4 (0.043)	Ref		
		AG	17 (0.172)	16 (0.172)	0.531 (0.134–2.113)	0.82	0.36571
		GG	74 (0.747)	73 (0.785)	0.507 (0.146–1.757)	1.18	0.27653
SFRP3	rs7775	Arg	54 (0.546)	2 (0.021)	Ref		
		Arg/Gly	3 (0.03)	29 (0.312)	0.004 (0.001–0.024)	67.63	**<0.0001**
		Gly	42 (0.424)	62 (0.667)	0.025 (0.006–0.109)	47.64	**<0.0001**
TCF7L2	rs12255372	GG	50 (0.505)	33 (0.355)	Ref		
		GT	43 (0.434)	45 (0.484)	0.631 (0.344–1.157)	2.23	0.13546
		TT	6 (0.061)	15 (0.161)	0.264 (0.093–0.750)	6.76	**0.00930**
LRP6	rs2075241	GG	62 (0.627)	67 (0.72)	Ref		
		GC	31 (0.313)	15 (0.226)	1.595 (0.830–3.064)	1.98	0.15936
		CC	6 (0.06)	5 (0.054)	1.297 (0.377–4.464)	0.17	0.67961
LRP6	rs2284396	TT	56 (0.566)	63 (0.678)	Ref		
		TC	32 (0.323)	21 (0.226)	1.714 (0.888–3.309)	2.60	0.10665
		CC	11 (0.111)	9 (0.096)	1.375 (0.531–3.561)	0.43	0.51078

BC. Breast Cancer; OR 95% CI. Odds Ratio and 95% Confidence Interval.

* P<0.05 was considered significant and are depicted in bold.

The heterozygous ‘AG’ genotype for the β-catenin SNP rs4135385 posed approximately two fold higher risk for developing breast cancers compared to women with homozygous ‘AA’ genotype (OR, 1.968; CI, 1.005–3.854; p = 0.04649). Genetic variants in AXIN2, DKK3, SFRP3 and TCF7L2 were associated with reduced risk of breast cancer. Compared to the homozygous genotype ‘AA’ in the AXIN2 rs3923087, the heterozygotes ‘AG’ showed about 2.7 fold decrease risk of developing cancer (OR, 0.373; CI, 0.193–0.720; p = 0.00298). The homozygosity of the minor allele in the DKK3 gene also provided modest protection against breast cancer (OR, 0.424; CI, 0.183–0.983; p = 0.04333). A strong protective association was observed between SNPs rs7775 and rs12255372 in SFRP3 and TCF7L2, respectively with breast cancer. The distribution of the three genotypes of SFRP3 as Arg and Gly homozygotes or Arg/Gly heterozygotes were significantly different in normal healthy individuals compared to that of breast cancer cases (χ^2^ = 73.1, df  = 2, p<0.0001). Furthermore, a significantly higher proportion of women with Arg/Arg to Gly/Gly homozygosity was observed in breast cancer cases compared to healthy individuals (χ^2^ = 47.64, df  = 1, p<0.0001). Women with genotype encoding Gly homozygosity were at 40 fold reduced risk of developing breast cancer compared to those having Arg homozygosity (OR, 0.025; CI, 0.006–0.109). Similar protection against breast cancer was observed for women with heterozygous Arg/Gly as well (OR, 67.63; CI, 0.001–0.024). It was noted that the minor allele (T) homozygotes for the TCF7L2 rs12255372 conferred 3.8 fold reduced risk for the development of breast cancers compared to women having major allele (G) homozygosity (OR, 0.264; CI, 0.093–0.750). We did not observe statistically significant association with the risk of developing breast cancer for the SNPs in APC, DKK4 and LRP6 as well as some of the SNPs in β-catenin (rs13072632), AXIN2 (rs4791171, rs11079571 and rs3923086) and SFRP3 (rs288326) in the overall study population.

### Effect of age of onset and ER status on the association of SNPs with breast cancer

In Saudi Arabian patients, the median age of onset of breast cancer is 47 years, substantially lower than 62 years observed in the United States [2], [3]. To evaluate the association of the analyzed SNPs with the young age at diagnosis of breast cancer, we stratified the patients as ≤43 (n = 38) or >43 (n = 61) years of age. The genotype distributions for the individual SNP along with the statistical analysis are shown in tables 3 and 4. Interestingly, only AXIN2 rs3923086 which did not show any association in the overall study or in the patient group with age >43 years indicated a protective influence on patients ≤43 years (Table 3). It was noted that, women aged ≤43 years with GG homozygosity were at 3.6 fold lower risk for developing breast cancer compared to those having the ancestral T allele homozygosity (OR, 0.274; CI, 0.099–0.758). Further, SNPs in β-catenin rs4135385 was associated with increased risk (AG genotype; OR, 3.03) and TCF7L2 rs12255372 was associated with decreased risk of breast cancer (TT genotype; OR, 0.213) in older age group patients (>43 years) similar to that observed in the overall study population. The stratification based on age of onset of the disease did not change the protective association of SNPs in AXIN2 rs3923087 and SFRP3 rs7775 as was observed in the overall study. Although the decreased risk of breast cancer with GG homozygosity compared with AA homozygosity in the AXIN2 rs3923087 was observed only in the younger patient cohort, the heterozygous AG genotype was associated with reduced risk in both the age groups as well as in the overall study. The SNP in the DKK3 gene that exhibited protective association in the overall study did not show any association when patients were categorized based on the age of onset of breast cancer.

**Table 3:** Comparison of genotype frequencies of SNPs in Wnt pathway genes between early on-set Breast cancers (patients age ≤43 years) versus controls.

Gene	SNP ID	Genotype	BC (≤43y) (n = 38)	Controls (n = 93)	OR (95% CI)	χ^2^ -Value	P*- Value
APC	Rs459552	Val	24 (0.631)	60 (0.645)	Ref		
		Val/Asp	12 (0.316)	31 (0.333)	0.968 (0 .427–2.192)	0.01	0.93733
		Asp	2 (0.053)	2 (0.022)	2.500 (0.333–18.777)	0 .84	0.35875
APC	Rs454886	TT	5 (0.131)	13 (0.14)	Ref		
		CT	12 (0.316)	33 (0.355)	0.945 (0.278–3.218)	0.01	0.92848
		CC	21 (0.553)	47 (0.505)	1.162 (0.367–3.678)	0.07	0.79871
β-Catenin	Rs13072632	CC	4 (0.105)	10 (0.107)	Ref		
		CT	20 (0.526)	42 (0.452)	1.190 (0.332–4.264)	0.07	0.78867
		TT	14 (0.369)	41 (0.441)	0.854 (0.231–3.160)	0.06	0.81256
β-Catenin	Rs4135385	AA	30 (0.79)	72 (0.774)	Ref		
		AG	6 (0.158)	18 (0.193)	0.800 (0.289–2.213)	0 .19	0.66686
		GG	2 (0.052)	3 (0.033)	1.600 (0.254–10.066)	0.25	0.61364
AXIN2	Rs4791171	AA	10 (0.263)	22 (0.344)	Ref		
		AG	17 (0.447)	44 (0.473)	1.236 (0.501–3.054)	0.21	0.64528
		GG	11 (0.29)	17 (0.183)	2.071 (0.733–5.852)	1.92	0.16629
AXIN2	Rs11079571	GG	19 (0.50)	45 (0.484)	Ref		
		GA	15 (0.395)	37 (0.398)	0.960 (0.429–2.147)	0.01	0.92114
		AA	4 (0.105)	11 (0.118)	0.861 (0.243–3.048)	0.05	0.81669
AXIN2	Rs3923087	AA	20 (0.54)	24 (0.258)	Ref		
		AG	13 (0.351)	50 (0.538)	0.312 (0.133–0.731)	7.48	**0.00623**
		GG	4 (0.109)	19 (0.204)	0.253 (0.074–0.865)	5.17	**0.02292**
AXIN2	Rs3923086	TT	15 (0.395)	16 (0.172)	Ref		
		TG	14 (0.368)	42 (0.451)	0.356 (0.141–0.900)	4.91	**0.02668**
		GG	9 (0.237)	35 (0.377)	0.274 (0.099–0.758)	6.52	**0.01066**
DKK3	Rs6485350	AA	19 (0.5)	28 (0.301)	Ref		
		AG	14 (0.369)	43 (0.462)	0.480 (0.207–1.110)	2.99	0.08364
		GG	5 (0.131)	22 (0.237)	0.335 (0 .108–1.039)	3.76	0.05263
DKK4	Rs3763511	CC	24 (0.632)	70 (0.753)	Ref		
		CT	13 (0.342)	23 (0.247)	1.649 (0.724–3.755)	1.43	0.23163
		TT	1 (0.026)	0	8.633 (0.340–218.9)	2.83	0.09253
SFRP3	Rs288326	AA	3 (0.079)	4 (0.043)	Ref		
		AG	7 (0.184)	16 (0.172)	0.583 (0.102–3.325)	0 .37	0.54155
		GG	28 (0.737)	73 (0.785)	0.511 (0.108–2.431)	0.73	0.39201
SFRP3	Rs7775	Arg	22 (0.579)	2 (0.021)	Ref		
		Arg/Gly	0	29 (0.312)	0.002 (0.000–0.041)	45.45	**1.567e-11**
		Gly	16 (0.421)	62 (0.667)	0.023 (0.005–0.110)	39.75	**2.886e-10**
TCF7L2	Rs12255372	GG	19 (0.5)	33 (0.355)	Ref		
		GT	16 (0.421)	45 (0.484)	0.618 (0.277–1.378)	1.40	0.23751
		TT	3 (0.079)	15 (0.161)	0.347 (0.089–1.356)	2 .45	0.11752
LRP6	Rs2075241	GG	26 (0.684)	67 (0.72)	Ref		
		GC	10 (0.263)	15 (0.226)	1.227 (0.510–2.955)	0.21	0.64775
		CC	2 (0.053)	5 (0.054)	1.031 (0.188–5.649)	0.00	0.97215
LRP6	Rs2284396	TT	24 (0.631)	63 (0.678)	Ref		
		TC	11 (0.29)	21 (0.226)	1.375 (0.577–3.275)	0.52	0.47112
		CC	3 (0.079)	9 (0.096)	0.875 (0.218–3.508)	0.04	0.85043

BC. Breast Cancer; OR 95% CI. Odds Ratio and 95% Confidence Interval.

* P<0.05 was considered significant and are depicted in bold.

**Table 4:** Comparison of genotype frequencies of SNPs in Wnt pathway genes between Breast cancers (patients age >43 years) versus controls.

Gene	SNP ID	Genotype	BC (>43y) (n = 61)	Controls (n = 93)	OR (95% CI)	χ^2^ -Value	P*- Value
APC	Rs459552	Val	43 (0.705)	60 (0.645)	Ref		
		Val/Asp	18 (0.295)	31 (0.333)	0.810 (0.402–1.632)	0.35	0.55566
		Asp	0	2 (0.022)	0.278 (0.013–5.940)	1.41	0.23439
APC	Rs454886	TT	9 (0.148)	13 (0.14)	Ref		
		CT	32 (0.524)	33 (0.355)	1.401 (0.526–3.729)	0.46	0.49912
		CC	20 (0.328)	47 (0.505)	0.615 (0.227–1.668)	0.92	0.33696
β-Catenin	Rs13072632	CC	5 (0.082)	10 (0.107)	Ref		
		CT	26 (0.426)	42 (0.452)	1.238 (0.381–4.028)	0.13	0.72241
		TT	30 (0.492)	41 (0.441)	1.463 (0.453–4.726)	0.41	0.52284
β-Catenin	Rs4135385	AA	33 (0.525)	72 (0.774)	Ref		
		AG	25 (0.426)	18 (0.193)	3.030 (1.456–6.305)	9.13	**0.00251**
		GG	3 (0.049)	3 (0.033)	2.182 (0.418–11.389)	0.89	0.34459
AXIN2	Rs4791171	AA	24 (0.393)	22 (0.344)	Ref		
		AG	27 (0.443)	44 (0.473)	0.818 (0.401–1.671)	0.30	0 .58152
		GG	10 (0.164)	17 (0.183)	0.784 (0.305–2.015)	0.26	0.61346
AXIN2	Rs11079571	GG	36 (0.59)	45 (0.484)	Ref		
		GA	22 (0.361)	37 (0.398)	0.743 (0.374–1.476)	0.72	0.39600
		AA	3 (0.049)	11 (0.118)	0.341 (0.088–1.315)	2.61	0.10599
AXIN2	Rs3923087	AA	25 (0.41)	24 (0.258)	Ref		
		AG	22 (0.36)	50 (0.538)	0.422 (0.199–0.896)	5.14	**0.02337**
		GG	14 (0.23)	19 (0.204)	0.707 (0.291–1.721)	0.58	0.44465
AXIN2	Rs3923086	TT	12 (0.197)	16 (0.172)	Ref		
		TG	27 (0.443)	42 (0.451)	0.857 (0.352–2.089)	0.12	0.73445
		GG	22 (0.36)	35 (0.377)	0.838 (0.334–2.101)	0.14	0.70628
DKK3	Rs6485350	AA	20 (0.328)	28 (0.301)	Ref		
		AG	33 (0.541)	43 (0.462)	1.074 (0.517–2.233)	0.04	0.84747
		GG	8 (0.131)	22 (0.237)	0.509 (0.189–1.373)	1.81	0.17909
DKK4	Rs3763511	CC	46 (0.754)	70 (0.753)	Ref		
		CT	13 (0.213)	23 (0.247)	0.860 (0.396–1.867)	0.15	0.70307
		TT	2 (0.0328)	0	7.581 (0.356–161.49)	2.97	0.08498
SFRP3	Rs288326	AA	5 (0.082)	4 (0.043)	Ref		
		AG	10 (0.164)	16 (0.172)	0.500 (0.108–2.318)	0.80	0.37178
		GG	46 (0.754)	73 (0.785)	0.504 (0.129–1.975)	1.00	0.31803
SFRP3	Rs7775	Arg	32 (0.525)	2 (0.021)	Ref		
		Arg/Gly	3 (0.049)	29 (0.312)	0.006 (0 .001–0.041)	47.53	**5.423e-12**
		Gly	26 (0.426)	62 (0.667)	0.026 (0.006–0.117)	41.00	**1.521e-10**
TCF7L2	Rs12255372	GG	31 (0.508)	33 (0.355)	Ref		
		GT	27 (0.443)	45 (0.484)	0.639 (0.322–1.266)	1.66	0.19799
		TT	3 (0.049)	15 (0.161)	0.213 (0.056–0.807)	5.84	**0.01564**
LRP6	Rs2075241	GG	36 (0.59)	67 (0.72)	Ref		
		GC	21 (0.344)	15 (0.226)	1.861 (0.899–3.854)	2.83	0.09241
		CC	4 (0.066)	5 (0.054)	1.489 (0.376–5.893)	0.32	0.56869
LRP6	Rs2284396	TT	32 (0.525)	63 (0.678)	Ref		
		TC	21 (0.344)	21 (0.226)	1.969 (0.940–4.124)	3.27	0.07062
		CC	8 (0.131)	9 (0.096)	1.750 (0.617–4.966)	1.12	0.28917

BC. Breast Cancer; OR 95% CI. Odds Ratio and 95% Confidence Interval.

* P<0.05 was considered significant and are depicted in bold.

We conducted the association of breast cancer risk with the individual SNPs based on the estrogen receptor (ER) status of the tumors. The genotype distribution in the ER + (n = 53) and ER- (n = 43) groups were separately compared with the genotype frequency in the same group of normal healthy women (n = 93) (Tables 5 and 6). Interestingly, homozygosity of the minor allele (T) in the DKK4 gene (rs3763511) posed significantly increased risk of ER- breast cancer (OR, 16.7; CI, 0.838–334.06; p = 0.00932). This association was not observed in the ER + category as well as in the overall study population. The AXIN2 rs3923087, DKK3 rs6485350 and the SFRP3 rs7775 that was significantly associated with decreased risk of breast cancer in the overall study population also exhibited significant protection for the ER + as well as ER- group (Tables 5 and 6). A nominal but significant increase in the risk of breast cancer was associated with the β-catenin rs4135385 in the overall study, however, such an association was not observed in either the ER + or ER- group. Additionally, the TCF7L2 rs12255372 displayed significant association with decreased risk of breast cancer in ER- (OR, 0.105, CI, 0.013–0.853, p = 0.01351) category similar to that in the overall population but not in the ER + cohort. As in the overall study, none of the other SNPs except those mentioned above displayed significant association with the risk of breast cancer in the ER + and ER- subgroup analyses.

**Table 5:** Comparison of genotype frequencies of SNPs in Wnt pathway genes between ER + breast cancers versus controls.

Gene	SNP ID	Genotype	BC (ER+) (n = 53)	Controls (n = 93)	OR (95% CI)	χ^2^ -Value	P*- Value
APC	Rs459552	Val	37 (0.699)	60 (0.645)	Ref		
		Val/Asp	16 (0.301)	31 (0.333)	0.837 (0.404–1.736)	0.23	0.63226
		Asp	0	2 (0.022)	0.323 (0.015–6.906)	1.22	0.26972
APC	Rs454886	TT	8 (0.151)	13 (0.14)	Ref		
		CT	23 (0.434)	33 (0.355)	1.133 (0.405–3.170)	0.06	0.81253
		CC	22 (0.415)	47 (0.505)	0.761 (0.275–2.101)	0.28	0.59703
β-Catenin	Rs13072632	CC	4 (0.075)	10 (0.107)	Ref		
		CT	25 (0.472)	42 (0.452)	1.488 (0.422–5.250)	0.39	0.53492
		TT	24 (0.453)	41 (0.441)	1.463 (0.413–5.181)	0.35	0.55347
β-Catenin	Rs4135385	AA	35 (0.66)	72 (0.774)	Ref		
		AG	16 (0.302)	18 (0.193)	1.829 (0.834–4.010)	2.30	0.12930
		GG	2 (0.038)	3 (0.033)	1.371 (0.219–8.585)	0.11	0.73481
AXIN2	Rs4791171	AA	14 (0.264)	22 (0.344)	Ref		
		AG	28 (0.528)	44 (0.473)	1.455 (0.662–3.194)	0.88	0 .34955
		GG	11 (0.208)	17 (0.183)	1.479 (0.552–3.959)	0.61	0.43497
AXIN2	Rs11079571	GG	29 (0.547)	45 (0.484)	Ref		
		GA	19 (0.359)	37 (0.398)	0.797 (0.386–1.643)	0.38	0.53826
		AA	5 (0.094)	11 (0.118)	0.705 (0.222–2.240)	0.35	0.55255
AXIN2	Rs3923087	AA	27 (0.510)	24 (0.258)	Ref		
		AG	19 (0.358)	50 (0.538)	0.338 (0.158–0.724)	8.01	**0.00466**
		GG	7 (0.132)	19 (0.204)	0.327 (0.117–0.914)	4.73	**0.02968**
AXIN2	Rs3923086	TT	16 (0.302)	16 (0.172)	Ref		
		TG	21 (0.396)	42 (0.451)	0.500 (0.210–1.192)	2.48	0.11537
		GG	16 (0.302)	35 (0.377)	0.457 (0.184–1.137)	2.88	0.08969
DKK3	Rs6485350	AA	17 (0.321)	28 (0.301)	Ref		
		AG	32 (0.604)	43 (0.462)	1.226 (0.575–2.612)	0.28	0.59786
		GG	4 (0.075)	22 (0.237)	0.299 (0.088–1.018)	3.97	**0.04639**
DKK4	Rs3763511	CC	38 (0.717)	70 (0.753)	Ref		
		CT	15 (0.283)	23 (0.247)	1.201 (0.561–2.571)	0.22	0.63633
		TT	0	0	1.831 (0.036–94.114)	0.999	1.00000
SFRP3	Rs288326	AA	5 (0.094)	4 (0.043)	Ref		
		AG	9 (0.17)	16 (0.172)	0.450 (0.096–2.115)	1.04	0.30670
		GG	39 (0.736)	73 (0.785)	0.427 (0.108–1.684)	1.55	0.21348
SFRP3	Rs7775	Arg	26 (0.49)	2 (0.021)	Ref		
		Arg/Gly	2 (0.038)	29 (0.312)	0.005 (0.001–0.040)	44.05	**<0.000001**
		Gly	25 (0.475)	62 (0.667)	0.031 (0.007–0.141)	35.29	**<0.000001**
TCF7L2	Rs12255372	GG	27 (0.510)	33 (0.355)	Ref		
		GT	22 (0.415)	45 (0.484)	0.598 (0.291–1.228)	1.98	0.15976
		TT	4 (0.075)	15 (0.161)	0.326 (0.097–1.098)	3.47	0.06246
LRP6	Rs2075241	GG	34 (0.642)	67 (0.72)	Ref		
		GC	15 (0.302)	15 (0.226)	1.501 (0.695–3.244)	1.08	0.29967
		CC	3 (0.056)	5 (0.054)	1.182 (0.267–5.244)	0.05	0.82541
LRP6	Rs2284396	TT	30 (0.566)	63 (0.678)	Ref		
		TC	20 (0.378)	21 (0.226)	2.000 (0.944–4.238)	3.32	0.06840
		CC	3 (0.056)	9 (0.096)	0.700 (0.177–2.774)	0.26	0.61025

BC. Breast Cancer; ER+. Estrogen Receptor positive; OR 95% CI. Odds Ratio and 95% Confidence Interval.

* P<0.05 was considered significant and are depicted in bold.

**Table 6:** Comparison of genotype frequencies of SNPs in Wnt pathway genes between ER- breast cancers versus controls.

Gene	SNP ID	Genotype	BC (ER-) (n = 43)	Controls (n = 93)	OR (95% CI)	χ^2^ -Value	P*- Value
APC	rs459552	Val	28 (0.651)	60 (0.645)	Ref		
		Val/Asp	13 (0.302)	31 (0.333)	0.899 (0.409–1.976)	0.07	0.79024
		Asp	2 (0.047)	2 (0.022)	2.143 (0.287–16.003)	0.58	0.44806
APC	rs454886	TT	6 (0.14)	13 (0.14)	Ref		
		CT	19 (0.442)	33 (0.355)	1.247 (0.407–3.823)	0.15	0.69850
		CC	18 (0.418)	47 (0.505)	0.830 (0.274–2.516)	0.11	0.74148
β-Catenin	rs13072632	CC	5 (0.116)	10 (0.107)	Ref		
		CT	19 (0.442)	42 (0.452)	0.905 (0.272–3.012)	0.03	0.87039
		TT	19 (0.442)	41 (0.441)	0.927 (0.278–3.088)	0.02	0.90150
β-Catenin	rs4135385	AA	27 (0.628)	72 (0.774)	Ref		
		AG	13 (0.302)	18 (0.193)	1.926 (0.832–4.458)	2.38	0.12269
		GG	3 (0.07)	3 (0.033)	2.667 (0.507–14.029)	1.43	0.23147
AXIN2	rs4791171	AA	19 (0.442)	22 (0.344)	Ref		
		AG	14 (0.326)	44 (0.473)	0.536 (0.234–1.225)	2.21	0.13695
		GG	10 (0.232)	17 (0.183)	0.991 (0.377–2.602)	0.00	0.98489
AXIN2	rs11079571	GG	25 (0.582)	45 (0.484)	Ref		
		GA	16 (0.372)	37 (0.398)	0.778 (0.363–1.670)	0.41	0.51973
		AA	2 (0.046)	11 (0.118)	0.327 (0.067–1.595)	2.06	0.15076
AXIN2	rs3923087	AA	17 (0.405)	24 (0.258)	Ref		
		AG	15 (0.357)	50 (0.538)	0.424 (0.181–0.989)	4.03	**0.04462**
		GG	10 (0.238)	19 (0.204)	0.743 (0.277–1.992)	0.35	0.55449
AXIN2	rs3923086	TT	10 (0.232)	16 (0.172)	Ref		
		TG	19 (0.442)	42 (0.451)	0.724 (0.278–1.887)	0.44	0.50768
		GG	14 (0.326)	35 (0.377)	0.640 (0.234–1.747)	0.76	0.38221
DKK3	rs6485350	AA	21 (0.488)	28 (0.301)	Ref		
		AG	13 (0.302)	43 (0.462)	0.403 (0.174–0.933)	4.61	**0.03188**
		GG	9 (0.210)	22 (0.237)	0.545 (0.209–1.425)	1.55	0.21337
DKK4	rs3763511	CC	29 (0.674)	70 (0.753)	Ref		
		CT	11 (0.256)	23 (0.247)	1.154 (0.499–2.671)	0.11	0.73711
		TT	3 (0.07)	0	16.729 (0.838–334.06)	6.76	**0.00932**
SFRP3	rs288326	AA	3 (0.07)	4 (0.043)	Ref		
		AG	8 (0.186)	16 (0.172)	0.667 (0.119–3.726)	0.21	0.64309
		GG	32 (0.744)	73 (0.785)	0.584 (0.124–2.764)	0.47	0.49380
SFRP3	rs7775	Arg	27 (0.628)	2 (0.021)	Ref		
		Arg/Gly	1 (0.023)	29 (0.312)	0.003 (0.000–0.030)	47.66	**5.080e-12**
		Gly	15 (0.349)	62 (0.667)	0.018 (0.004–0.084)	47.73	**4.891e-12**
TCF7L2	rs12255372	GG	21 (0.488)	33 (0.355)	Ref		
		GT	21 (0.488)	45 (0.484)	0.733 (0.345–1.558)	0.65	0.41916
		TT	1 (0.024)	15 (0.161)	0.105 (0.013–0.853)	6.10	**0.01351**
LRP6	rs2075241	GG	25 (0.581)	67 (0.72)	Ref		
		GC	15 (0.349)	15 (0.226)	1.914 (0.855–4.287)	2.53	0.11173
		CC	3 (0.070)	5 (0.054)	1.608 (0.358–7.230)	0.39	0.53268
LRP6	rs2284396	TT	26 (0.605)	63 (0.678)	Ref		
		TC	10 (0.232)	21 (0.226)	1.154 (0.478–2.784)	0.10	0.75005
		CC	7 (0.163)	9 (0.096)	1.885 (0.635–5.596)	1.33	0.24885

BC. Breast Cancer; ER-. Estrogen Receptor negative; OR 95% CI. Odds Ratio and 95% Confidence Interval.

* P<0.05 was considered significant and are depicted in bold.

## Discussion

The Wnt signaling pathway plays an essential role during embryonic development and has been causally linked to several malignancies including breast cancer [13], [14], [19]. While genetic alterations of Wnt pathway components, such as APC, β-catenin, axin and TCF7L2 are implicated in colorectal cancers, melanoma, gastric and hepatocellular carcinomas, they are rarely associated with breast cancer [12], [14], [20]–[25]. Elevated levels of nuclear and/or cytoplasmic β-catenin have been reported in a large proportion of breast cancers, suggesting activation of the Wnt signaling pathway due to alternate mechanisms such as autocrine signaling and reduced expression of the soluble extracellular Wnt inhibitors [14], [17], [26]–[28]. In this study, we took a pathway based candidate gene approach to identify breast cancer risk association with genetic variants in Wnt signaling pathway genes in Saudi women. To our knowledge this is the first study to utilize such an approach to link SNPs in Wnt pathway genes with predisposition to breast cancers in Saudi Arabian population. An earlier study utilizing similar approach with different sets of SNPs and genes in the Wnt signaling pathway did not find any association with colorectal cancers in Spanish population [29]. Only the rs459552 in APC gene which did not show any association with breast cancer in our analysis was common with the study reported by Fernandez-Rozadilla et al. Five of the 15 SNPs that were examined in this study showed significant association with breast cancer. Four of the 5 significantly associated SNPs were in the noncoding region and only SFRP3 rs7775 was in the exon that codes for either Arg (**C**GC) or Gly (**G**GC). The product of SFRP3 gene is a natural inhibitor of the Wnt signaling pathway that functions by sequestration of the Wnt ligands and thus avoiding its interaction with its receptor [30]. While the C allele coding for Arg is the ancestral and the G allele that codes for Gly is the minor allele, we observed that women with Arg/Gly and Gly/Gly genotypes were at highly significant reduced risk of developing breast cancer compared to women with Arg/Arg genotype (Arg/Arg vs Arg/Gly, OR, 0.004; CI, 0.001–0.024; p<0.0001; Arg/Arg vs Gly/Gly, OR, 0.025; CI, 0.006–0.109; p<0.0001). Loughlin and coinvestigators found that the G allele of rs7775 was significantly associated with osteoarthritis in females [31]. However, no study pertaining the analysis of this SNP in cancers has been reported. Our finding of the strong protection conferred by the CG as well as GG genotypes of rs7775 against breast cancers though in a small population size is significant and merits examination of this SNP in larger studies of different ethnic groups.

TCF7L2 is a component of Wnt/β-catenin signaling pathway which is implicated in several human cancers including breast cancers. Binding of β-catenin to TCF7L2 in the nucleus converts it to transcriptional activator resulting in the expression of target genes involved in cellular proliferation, inhibition of apoptosis, invasion and metastasis. TCF7L2 gene variant rs12255372 has been reported to be associated with risk of Type 2 diabetes in different populations [32], [33]. Few studies have investigated the association of rs12255372 and the risk of breast cancers with contradictory results [34]–[37]. We investigated the risk associated with this polymorphism in Saudi Arabian population and found an inverse association of the rare variant genotype TT with the risk of breast cancer. Thus, our analysis suggest a statistically significant protection against breast cancer for women with TT genotype compared with the GG genotype in TCF7L2 gene (OR, 0.264; CI, 0.093–0.750; χ^2^ = 6.76, df  = 1; p = 0.0093). This association was also statistically significant for patients who were >43 years of age and whose tumors were ER negative. The differences observed between our results and other breast cancer studies could be due to various possible factors including ethnicity, sample size and different histopathological tumor types included in the study. Our results are consistent with the observation made by Hazra et al, who reported an inverse association of the TT genotype of rs12255372 in the TCF7L2 gene with colorectal cancers [38].

Of the two SNPs in the β-catenin gene that were analyzed, significant association was observed for rs4135385 with breast cancer while the other SNP rs13072632 was not associated. A significantly higher frequency of heterozygous genotype AG was represented in the breast cancer group (0.313) compared to control group (0.193) (χ^2^ = 3.96, df  = 1; p = 0.04649). Thus, women with AG genotype were at approximately two fold higher risk of developing breast cancer compared with those having AA genotype (OR, 1.968; CI, 1.005–3.854). Wang et al investigated the rs4135385 and identified significant association of this SNP with gastric cancer risk in Chinese population [39]. However, the percent distribution of the three genotypes (AA/AG/GG – Cases; 8.9/43.6/47.5; Controls; 7.6/38.1/54.3) was very different to that observed in Saudi women in our study (AA/AG/GG – Cases; 63.7/31.3/5.0; Controls; 77.4/19.3/3.3). The ancestral allele A is more represented in our population compared to the Chinese population studied by Wang et al [39]. This difference could be due to the ethnic diversity between the two populations. Given the fact that β-catenin mutations are extremely rare, it would be highly desirable to find variants in the gene that could be linked to breast cancers. Thus, larger studies in Saudi population as well as other ethnicities are required to confirm the association of rs4135385 with the risk of breast cancer observed in our analysis.

The homologous proteins AXIN1 and AXIN2 are components of the β-catenin destruction complex and thus, act as tumor suppressors by negatively regulating the Wnt signaling pathway [40], [41]. Although, somatic mutations and epigenetic silencing of AXIN2 gene is uncommon in breast cancers, it has been reported in colorectal and lung cancers, respectively [42], [43]. We investigated four SNPs in AXIN2 gene to identify predisposition of breast cancers to any of these genetic variants. In the overall analysis only the rs3923087 exhibited significant association with breast cancer (Table 2). The heterozygous genotype AG of rs3923087 conferred significant protection against the risk of breast cancer compared to the AA genotype (OR, 0.373; CI, 0.193–0.720). This association was persistent even after the stratification of the population based on ER status and age of on-set of breast cancers (Tables 3–6). Additionally, compared to AA, the homozygous GG genotype of rs3923087 also showed significant protection against the ER + (OR, 0.327; CI, 0.117–0.914) and early on-set (OR, 0.253; CI, 0.074–0.865) of breast cancers. Moreover, a significant protective association was observed with the rs3923086 in only the younger patient cohort whose age was ≤43 years (Table 3). Wang et al examined a number of SNPs in the AXIN2 gene including the four variants in this study and reported a significantly elevated risk with premenopausal breast cancers [44]. The discrepancy between our results and Wang et al could be due to genetic variations in the population and the difference in the pathological types of tumors used in the study. Also, the sample size in this study is relatively smaller compared to Wang et al [44]. In a Japanese population a SNP at codon 50 of the AXIN2 gene was shown to have a significantly reduced risk of developing lung cancer while association with head and neck and colorectal cancer was not observed [45].

The DKK gene family encodes secreted proteins that antagonizes the Wnt signaling pathway whose expression has been downregulated in several different cancers including breast and thus, act as putative tumor suppressor [28], [46]. We examined rs6485350 in DKK3 gene for its association with the risk of breast cancer. This is the only SNP reported for DKK3 gene until the writing of this manuscript in the SNP500Cancer project accessed from the http://variantgps.nci.nih.gov/cgfseq/pages/snp500.do website. Women with GG genotype were at approximately 2-fold reduced risk for developing breast cancer compared to those having AA genotype. When the tumors were grouped based on the ER status, homozygous GG genotype conferred protection against the risk of developing ER + tumors (OR, 0.299; CI, 0.088–1.018) while the heterozygous AG genotype showed protective association against ER- breast cancers (OR, 0.403; CI, 0.174–0.933). No statistical significant association was observed for this SNP in the DKK3 gene with the age of on-set of breast cancers. Additionally, the rs3763511 in DKK4 that did not display any association in the overall as well as age related analysis was strongly associated with increased risk of ER- breast cancer. To our knowledge this is the first study to report an association of rs6485350 in the DKK3 and rs3763511 in DKK4 genes with breast cancer.

Thus, in this preliminary study, we undertook pathway based candidate gene approach and identified five SNPs in critical genes involved in Wnt signaling to be significantly associated with breast cancers. Except the SNP in β-catenin gene which was associated with increased risk of breast cancers, the other four SNPs in AXIN2, DKK3, SFRP3 and TCF7L2 genes were associated in a protective manner. Though, the population size in our study is small, the findings are significant that need to be confirmed in larger and ethnically different groups for the identified potential markers to be used for breast cancer screening. Moreover, a larger patient cohort stratified based on the age of on-set of the disease might provide the influence of genetic variants in the Wnt signaling pathway genes on the early on-set of breast cancers typically observed in Saudi women.
